# Isolation and characterization of novel *Klebsiella* phages from Benin and their antibiofilm activities on multidrug resistant and hypervirulent *Klebsiella pneumoniae* strains

**DOI:** 10.3389/fmicb.2026.1948708

**Published:** 2026-10-26

**Authors:** Alidehou Jerrold Agbankpe, Kafayath Fabiyi, Yaovi M. Gildas Hounmanou, Gracia Ruth Nougbologni, Roubaya Balarabe, Slawomir Michniewski, Jules Beau-Gard Hougbenou, Esther Deguenon, Victorien Dougnon, Honoré Bankolé, Lamine Baba-Moussa, Rashid Nazir, Martha R. J. Clokie

**Affiliations:** 1 Bacteriology Research Unit, Institut Pasteur de Guinée, Conakry, Guinea; 2 Becky Mayer Centre for Phage Research, Division of Microbiology and Infection, University of Leicester, Leicester, United Kingdom; 3 Research Unit in Applied Microbiology and Pharmacology of Natural Substances, Research Laboratory in Applied Biology, Polytechnic School of Abomey-Calavi, University of Abomey-Calavi, Cotonou, Benin; 4 Research Center for Global Health Action on Climate Change, Gender Equity, and Emerging Diseases, Pahou, Benin; 5 Stellenbosch Institute for Advanced Study (STIAS), Wallenberg Research Centre at Stellenbosch University, Stellenbosch, South Africa; 6 Department of Veterinary and Animal Sciences, Faculty of Health and Medical Sciences, University of Copenhagen, Frederiksberg, Denmark; 7 Laboratory of Biology and Molecular Typing in Microbiology, Faculty of Science and Technology, University of Abomey-Calavi, Cotonou, Benin; 8 Department of Environmental Sciences, COMSATS University Islamabad (CUI), Abbottabad Campus, Abbottabad, Pakistan

**Keywords:** antibiofilm activity, bacteriophages, Benin, hypervirulent *K. pneumoniae*, multidrug resistant, phage therapy

## Abstract

Hypervirulent *Klebsiella pneumoniae* represents a growing clinical threat, particularly where multidrug resistance limits treatment options. This study aims to characterize *Klebsiella* phages isolated in Benin and evaluate their efficacy in reducing bacterial cell growth, biofilm inhibition and biofilm disruption. To achieve this objective, we isolated *Klebsiella* phages by enriching samples of hospital effluent, pig droppings, and stool, using clinical multidrug resistant and hypervirulent *K. pneumoniae* strains as hosts. These phages were characterized using transmission electron microscopy, whole-genome sequencing, and biological assays. Then, we evaluated their antibiofilm activity and stability under harsh conditions (pH and temperature) *in vitro*. So, we report the isolation and characterization of three novel lytic phages from Benin (Kp1Bj_HH11_M23, Kp2Bj_LN294_M23, and Kp10Bj_LN54_14). These Myoviruses, belonging to the genera *Marfavirus* and *Jiaodavirus*, display broad host range activity against multidrug resistant and hypervirulent *K. pneumoniae* strains with various capsular serotypes including KL114, KL53, KL8, KL62, KL64, KL124, KL102, KL57, KL39, KL48, KL17, KL20, KL2, KL30, KL51, KL105, KL146, KL28, KL122, KL38, KL3, and KL163. Genomic analysis confirmed the absence of virulence and antimicrobial resistance genes. The phages exhibit rapid adsorption, short latency periods (5–10 min), and high burst sizes (119–2208 PFU/cell). All three phages have significantly biofilm disruption effect *in vitro* (Kp1Bj_HH11_M23, biofilm disruption rate of 98.46 %, *p*-value = 0.0170; Kp2Bj_LN294_M23, biofilm disruption rate of 65.56%, *p*-value < 0.0001; Kp10Bj_LN54_14, biofilm disruption rate of 99.96%, *p*-value = 0.003). Their stability across a wide range of temperatures and pH further supports their potential for therapeutic development. Together, these data highlight the value of locally sourced phages as candidates for tackling region-specific antimicrobial resistance challenges.

## 1 Introduction

Antibiotics have been essential as a first line of defense against pathogens, playing a crucial role in the fields of human and animal health (Manyi-Loh et al., 2018; Rahman et al., 2025). However, several decades since the introduction of antibiotics, the world is facing an uncertain situation in the advent of the post-antibiotic era. Basically, the uncontrolled use of antibiotics in human and veterinary medicine has amplified and accelerated the evolution of antibiotic resistance (Schooley et al., 2017). The problem of transmission and dissemination of resistance has been intensified by environmental contamination of resistant bacteria and resistance genes from people and animals (Carvalho et al., 2017). To this end, communities and hospitals feel invaded by the increasing spread of antibiotic-resistance bacteria is threatening our ability to treat serious infections and represents a significant public health problem globally (Salam et al., 2023). Gram-negative bacteria constitute a significant fraction of multidrug resistant pathogens, including *Klebsiella pneumoniae*, for which there is an urgent need to research and develop new treatments (World Health Organization, 2024).

*Klebsiella pneumoniae* infections are often considered one of the deadliest infections for infants, the elderly, and immuno-compromised patients. Despite the development of broad-spectrum antimicrobials, more than 50% of mortality rates have been recorded in individuals infected with *K. pneumoniae* (Bassetti et al., 2018; Fage et al., 2021). Antimicrobial resistance (AMR) in *K. pneumoniae* is a major clinical challenge, particularly with the emergence of strains combining multidrug resistance and hypervirulence (Arcari and Carattoli, 2023; Gan et al., 2025). In early 2024, the World Health Organization (WHO) reported the global emergence of *K. pneumoniae* ST23 strains carrying carbapenemase genes, which confer resistance to last-resort antibiotics (Abdel-Rahman et al., 2026). This coexistence of hypervirulence and multidrug resistance is associated with severe infections, high mortality, and limited treatment options, particularly in low- and middle-income countries (LMICs) (Kansal et al., 2026).

Recent studies in Benin highlight the alarming situation regarding antibiotics resistance (Yehouenou et al., 2020, Yehouenou et al., 2022; Ombelet et al., 2022; Sintondji et al., 2023, 2025). Data from these studies indicate a growing prevalence of extended-spectrum beta-lactamase (ESBL)-producing bacteria and the emergence of carbapenem-resistant in infections (surgical site infections, urinary tract infections, bloodstream infections) (Ombelet et al., 2022; Yehouenou et al., 2022; Dougnon et al., 2023; Sintondji et al., 2025). Among the bacteria isolated in these various studies, *K. pneumoniae* is one of the most prevalent. 77.6% of the bacteria isolated from cases of bloodstream infections recorded at a secondary hospital in northern Benin were *K. pneumoniae*, and all were multidrug resistant (Ombelet et al., 2022). Furthermore, 68.9% (95% CI, 62.4%–74.8%) of *K. pneumoniae* isolates associated with urinary tract infections in Benin between 2010 and 2022 were ESBL-producing, with a significant increase (from 0.9% to 14.5%) in carbapenem-resistant isolates (used as a last-resort treatment) (Rafetrarivony et al., 2026).

In light of the threat that multidrug resistant and hypervirulent *K. pneumoniae* strains pose to patient health, there has been a rise in research on phages and phage therapy as a promising alternative solution. Despite the public health burden that hypervirulent and multidrug resistant *K. pneumoniae* can pose in Benin, there is a lack of country-specific data on phages that target clinically relevant strains of *K. pneumoniae*. As agents of bacterial mortality, phages exert evolutionary pressure on their hosts. Therefore, local phages will have the advantage of infecting the diverse *K. pneumoniae* strains circulating in Benin and will serve as potential candidates for a therapy against infections caused by multidrug resistant and hypervirulent *K. pneumoniae* strains. The ability to form biofilms is one of the main virulence factors of *K. pneumoniae.* Biofilm formation plays a key role in the spread of hypervirulent *K. pneumoniae* strains, enhances resistance to antibiotics and the host’s immune responses, and makes infections more persistent and difficult to treat (Guerra et al., 2022). Phages represent one of the emerging approaches to overcoming this challenge. Several studies have demonstrated the *in vitro* efficacy of phage antibiofilm activity against multidrug resistant and hypervirulent *K. pneumoniae* strains. What about phages isolated locally in Benin? This study was therefore conducted to answer this question. Its objectives are the isolation, biological and genomic characterization, assessment of the host range, and evaluation of the antibiofilm activity of Beninese *Klebsiella* phages.

## 2 Material and methods

### 2.1 Bacterial strains

A collection of sixty clinical strains of hypervirulent and multidrug resistant *K. pneumoniae* were used in this study. The strains were obtained from the bacterial strain collections of the Becky Meyer Centre for Phage Research, University of Leicester, United Kingdom (UK) and the Research Unit in Applied Microbiology and Pharmacology of natural substances, University of Abomey-Calavi, Benin. These strains were isolated from clinical samples of urine (midstream or catheter specimen urine), blood, rectal swab, sputum and endotracheal secretions, and with a diversity of capsular loci (KL) (Supplementary Table 1). All *K. pneumoniae* strains were grown at 37 °C in either Luria-Bertani broth (LB-Thermo Fisher Scientific, UK) at 200 rpm or on LB 1.5% (w/v) agar plates and then used as hosts for phage isolation and host range evaluation.

### 2.2 Phage isolation, purification and visualization

Phages were isolated from 50 samples including 20 hospital effluents, 15 farmer’s stools and 15 pig droppings collected from pig farms located on Lake Nokoué in Benin (Supplementary Table 2) using *K. pneumoniae* host strains as previously described with slight modification (Haines et al., 2021). Samples (50 mL) were centrifuged at 4,200 × *g* for 10 min to remove debris and other undissolved particles, and the supernatant was filtered using a 0.45 μm filter. Filtrate (10 mL) was enriched by bacterial culture (100 μL) in LB broth (10 mL) supplemented with 40 μL CaCl_2_ (1M) and incubated overnight at 37 °C with shaking (200 rpm). The mixed culture solution was centrifuged at 4,200 × *g* for 15 min, and the supernatant was filtered using a 0.22 μm filter. Then, enriched filtrate (100 μL), bacterial culture (100 μL) and LB 0.6% (w/v) agar (3 mL) were poured onto a LB 1.5% (w/v) agar plate (90 mm petri dishes) and incubated overnight at 37 °C. Single plaques were picked and transferred to SM Buffer (500 μL). The mixture was shaken for 1h by inversion, centrifuged at 4,200 × *g* for 30 min and was filtered using a 0.22 μm filter.

Transmission electron microscopy (TEM) imaging for the phages was performed at a microscopy core facility of the University of Leicester, UK. 5 μL of phages were negatively stained with 1% (w/v) uranyl acetate on 3 mm carbon-coated copper grids and visualized with a JEM-1400 transmission electron microscope (JEOL UK Ltd., UK) with an accelerating voltage of 120kV. Digital images were collected using a Xarosa digital camera (EMSIS, Germany) with Radius software.

### 2.3 Titration, host range and efficiency of plaquing (EOP)

The titration of each phage was performed using the method previously described by El-Telbany et al. (2021). A serial dilution of each phage was performed in SM buffer (900 μL). Bacterial culture (250 μL, OD_600_ of ∼0.3) was added to 8 mL LB 0.6% (w/v) agar and poured onto LB 1.5% (w/c) agar plates (120 × 120 mm petri dishes) for bacteria lawn. Then, each dilution (10 μL) was spotted on LB agar plates and incubated at 37 °C overnight. The titer in plate-forming units per milliliter (PFU/mL) of each phage was calculated using the following formula: PFU/mL = (number of plaques × dilution factor)/volume spotted (mL).

Host range analysis was performed using a panel of fifty-eight *K. pneumoniae* strains (Supplementary Table 1) by spot test. Phage stock (10 μL) was spotted onto the plate (bacterial lawn) and incubated overnight at 37 °C. The plaques were graded to complete lysis and hazy lysis. Host-range testing was triplicate. Moreover, susceptibility of the tested strains to the phages was also performed using the efficiency of plaquing (EOP) method (El-Telbany et al., 2021; Kutter, 2009). Serial dilutions were made for each phage and 10 μL of the dilutions were spotted on lawns. Plaques were counted, and EOP was calculated using the following formula: average PFU/mL on tested strain/average PFU/mL on potential host.

### 2.4 Phage DNA extraction and sequencing

Phage DNA extraction was performed using the phenol-chloroform-isoamyl alcohol (PCI) method (Nale et al., 2015). Phage lysate (1 mL) was treated with 12.5 μL of 1M MgCl_2_, 10 μL of DNase I (30 mg/ml) and 1 μL of RNase A (100 mg/mL), followed by incubation for 30 min at room temperature. Proteins were removed and phage DNA was purified by two cycles of PCI extractions. Then, DNA was precipitated with 95% ethanol and 3M sodium acetate solution. After washing in 70% ethanol, the pellets were resuspended in 50 μL of water. DNA concentrations were quantified using a Qubit 2.0 fluorometer. Illumina sequencing libraries were prepared using the tagmentation-based and PCR-based Illumina DNA Prep kit and custom IDT 10bp unique dual indices (UDI) with a target insert size of 320 bp. No additional DNA fragmentation or size selection steps were performed. Illumina sequencing was performed on an Illumina NovaSeq 6000 sequencer for 151bp paired-end reads. Demultiplexing, quality control and adapter trimming was performed with bcl-convert (v4.1.5).

### 2.5 Bioinformatics analysis

#### 2.5.1 Phage genome identification and initial curation

Raw reads of phages were assembled with Spades v4.2.0 and added to a curated collection of 39 publicly available *K. pneumoniae* phage genomes. For each sample, assembled contigs in FASTA format were quality controlled with CheckV v1.0.3 and analyzed using VirSorter2 (v2.2.3), focusing on identification of dsDNA phages, with the dsDNAphage group enabled and a minimum contig length threshold of 1,500 bp. All resulting outputs were organized in structured directories for batch processing. To supplement VirSorter2 predictions and identify additional phage sequences, a complementary BLASTn-based filtering strategy was applied. All contigs were searched against the NCBI nucleotide (nt) database using BLASTn, with filters set to ≥ 75% identity, ≥ 25% query coverage, and E-value ≤ 1e-5. Resulting hits were retained if their annotations included the terms “phage”, “virus”, or “bacteriophage”. Corresponding contig headers were extracted using seqtk to generate refined FASTA files representing likely phage genomes.

#### 2.5.2 High-confidence dsDNA phage sequence selection

To increase the specificity of phage detection, candidate sequences were filtered for high-confidence double-stranded DNA (dsDNA) phages based on classification results from VirSorter2. Only contigs classified under the dsDNA phage group with a final_max_score of 1.0 were retained. Contig identifiers were mapped to headers in the final-viral-combined.fa files to extract full sequence entries. These sequences were then compiled into sample-specific FASTA files as well as individual contig files (*.fa) using a custom extraction pipeline. Curated phage sequences were consolidated across all samples into a central directory for downstream analyses. Empty files and low-quality outputs were removed to ensure a clean dataset for further processing.

#### 2.5.3 Phage genome quality assessment

All candidate phage genomes were evaluated using CheckV (v1.0.1) to assess genome completeness, contamination, and provirus content, using the CheckV database (v1.5). Quality summaries were parsed and centralized, and provirus sequences (viruses.fna) were extracted and renamed for standardized downstream analysis. Quast was then used to generate the overall assembly statistics on the final phage genomes retained for analysis.

#### 2.5.4 Phage genome annotation

High-quality phage genomes were annotated using two complementary pipelines:

- Cenote-Taker3 was applied for structural and functional annotation, with a focus on viral hallmark genes including those encoding capsid and tail proteins.
- Pharokka (v1.7.1) was used to annotate protein-coding genes across phage genomes based on the PHROGs and CARD databases. Annotation was performed on each phage genome individually, and results were stored in organized output directories. Visual summaries of the gene content were generated using pharokka_multiplotter.py.

#### 2.5.5 Taxonomic classification of phage genomes

To confirm and refine the taxonomic identities of the phage sequences, Kraken2 (v2.1.2) was used to classify all curated genomes against two viral databases. One Kraken2 run utilized a custom viral/phage-specific Kraken2 database, while another employed a standard RefSeq Kraken2 database. Classification reports were parsed to assess taxonomic diversity and ensure inclusion of known phage lineages.

#### 2.5.6 Phylogenetic analysis of phage genomes

To investigate the evolutionary relationships among phage genomes, we constructed phylogeny based on single nucleotide polymorphisms (SNPs) in accessory genome content. This analysis included the three reference genomes (from this study) and thirty-nine high-quality *K. pneumoniae* phage genomes downloaded from NCBI as publicly available genomes (Supplementary Table 3). We employed a pangenomic approach to explore gene content diversity among all forty-two phage genomes. Each genome was annotated using Pharokka, a tool specifically designed for phage genome annotation. The resulting GFF3 files were used as input for Roary, which clustered orthologous genes and produced a gene presence/absence matrix. A phylogenetic tree was subsequently inferred based on this binary gene content matrix. This approach allowed us to investigate evolutionary relationships shaped by gene gain and loss, capturing genomic variability not evident through SNP analysis alone and providing a broader view of the diversity and relatedness among phage genomes. Average Nucleotide Identity (ANI) analysis was performed between the three phages using fastANI V1.34.

#### 2.5.7 Comparative genome analysis

Pairwise whole-genome nucleotide comparisons were performed between three studied phages and their selected *Klebsiella* phage homologues (MG746602, MN044033, PQ133004, KY000080, NC_031087 and NC_031095) using BLASTn (NCBI BLAST+ v2.17.0) and fastANI V1.34. Graphical whole-genome comparisons were generated using Proksee, with each study phage used as the reference genome and its selected *Klebsiella* phages included as comparative tracks.

### 2.6 Optimal multiplicity of infection (MOI)

The MOI for each phage was determined using the method previously described by Han et al. (2023). Bacterial culture (150 μL) was mixed with different dilutions of phage suspension at MOIs of 0.001, 0.01, 0.1, 1, 10, and 100. The mixture was added to 5 ml of LB and incubated at 37 °C, 200 rpm for 18h. The culture was centrifuged at 4,200 × *g* for 10 min, and the supernatant was filtered using 0.22 μm filter. Then, phage titer under different MOI was determined using double layer agar method (Haines et al., 2021), and the highest phage titer was corresponded to the optimal MOI.

### 2.7 Adsorption assays and one-step growth curve

Adsorption assays were performed as previously described with some modification (Storms and Sauvageau, 2014). Bacterial culture (900 μL) was aliquoted to Eppendorf tubes labeled 0; 5; 10; 15; 20; 25; 30; and 60 min. Then, phage (100 μL) was added to each Eppendorf tubes at an optimal MOI and incubated at 37 °C. At each time point, mixture was removed and centrifuged at 12,000 rpm for 5 min, and the supernatant was filtered using 0.22 μm. Serial dilution of filtrate was carried out, and the titer of unadsorbed phage was determined using the double layer agar method.

One-step growth was carried out to understand the growth pattern of *Klebsiella* phage from Benin. Phage was added to bacterial culture at an optimal MOI. The mixture was incubated at 4 °C for 20 min to allow sufficient adsorption of phage to the host, and was centrifuged at 4,200 rpm for 10 min. Then, pellet (bacteria) were collected. Pellet was washed twice with SM buffer and resuspended in LB (10 mL). This suspension (1 mL) was aliquoted to Eppendorf tubes labeled 0; 5; 10; 15; 20; 30; 60; 90; and 120 min and was incubated at 37 °C, 200 rpm. At each time point, the mixture was removed and centrifuged at 12,000 rpm for 5 min and was filtered. Serial dilution of filtrate was carried out, and the titer of phage was determined using the double layer agar method. The number of viral particles produced from the infection of a single cell, called the burst size, was calculated using the ratio of the post-lysis (plateau) titer to the titer during the latent period (Zhao et al., 2019).

### 2.8 Antibiofilm activities of phages against *K. pneumoniae* host strains

*Klebsiella pneumoniae* produces a capsular polysaccharide that can be released into the extracellular environment to form biofilms that can enhance host defense and tolerance against antimicrobials (Anderl et al., 2000). Planktonic cells killing was performed and the inhibition of biofilm formation of *K. pneumoniae* strains by phages isolates for 48 h as well. The experiment was carried out over 4 days. Day 0: Four 12-well plates were labeled P1, P2, P3 and P4, respectively. Bacterial culture (1 mL, OD_600_ of ∼0.3) was added to wells A1-2, B1-2 and C1-2. Then, LB broth (1 mL) was added to wells A4, B4 and C4, which served as negative controls. The four plates were incubated at 37 °C, 100 rpm for 24 h. Day 1: planktonic cultures (500 μL) were removed from plates P3 and P4, taking care to not disturb the biofilms. Each planktonic culture was replaced by LB broth (500 μL). Both plates were incubated again at 37 °C, 100 rpm for 24 h. All the planktonic cultures were removed from plates P1 and P2 and the wells were washed 3 times by gently adding and removing PBS (1 mL). Then, phage (500 μL) was added to wells A1, B1 and C1 (phage treated wells), and LB broth (500 μL) were added to wells A2, B2 and C2 (bacterial control), and 500 μL of LB broth to wells A4, B4 and C4 as well. Plates were incubated at 37 °C, 100 rpm for a further 24 h. Day 2: on plate P1, planktonic cultures (500 μL) was removed from each of the phage treated and bacterial control wells and put into Eppendorf tubes and was washed three times by centrifuging at 12,000 rpm for 15 min and resuspending with PBS (500 μL). The final pellet was resuspended with 1 ml of PBS and serial dilution was conducted and bacteria was counted (CFU/ml) by spotting the dilution on to LB 1.5% (w/c) agar. The plates were incubated at 37 °C for 24 h. For biofilms, the wells were washed three times by gently adding and aspirating PBS (1 mL). Then PBS was added, and the bottom of wells was scraped to resuspend the biofilm. Serial dilution of the biofilm culture was performed, and bacteria was counted (CFU/ml). For the P2 plate, the plankton cultures were aspirated, and the wells were washed three times by adding and gently aspirating PBS. Then, 500 μL of 0.1% filtered crystal violet (CV) was added to the wells and placed on a rocker for 15 min at room temperature. The wells were gently washed under the tap to remove any unbound CV. Ethanol (100%, 1 mL) was added, and the plate was incubated at room temperature for 10 min. The well content (200 μL) was transferred to a new 96-well plate and the OD_600nm_ was measured using the BMG Labtech SPECTROstar Omega (Sarstedt, Germany). Then, experiments carried out on plates P1, and P2 on Day 1 were repeated on plates P3 and P4. Day 3: The experiments carried out on plates P1, and P2 on Day 2 were repeated on plates P3 and P4.

### 2.9 Thermal and pH stability

In Eppendorf tubes labeled 4, 10, 20, 30, 40, 50, 60, 70 and 80 °C, phages (100 μL) were added to SM buffer (900 μL). Tubes identified as 4 and 10 °C were incubated in the refrigerator and those at 20, 30, 40, 50, 60, 70 and 80°C were incubated in thermomixers for 1 h. Then, tubes were brought to room temperature for 1 h on the bench, and serial dilution of each tube (100 μL) was carried out. Titer of phage was determined using the double layer agar method. For pH stability, SM buffer was adjusted by adding HCl or NaOH to obtain the desired pH using an automatic pH meter.

### 2.10 Ethical approval

The study was approved by the National Health Research Ethics Committee of Benin (N°67/MS/DC/SGM/DRFMT/CNERS/SA of 20th April 2021) and farmers provided written informed consent.

## 3 Results

### 3.1 Isolation and morphology of phages

The three phages were isolated from hospital effluents (Kp1Bj_HH11_M23), farmers’ stools (Kp10Bj_LN54_14) and pig droppings (Kp2Bj_LN294_M23) on multidrug resistant and hypervirulent *K. pneumoniae* (Supplementary Table 2). Success rate for isolating these phages from the samples was 15% (*n* = 3) for hospital effluents, 13.33% (*n* = 2) for pig droppings, and 6.66% (*n* = 1) for stools. The phages Kp1Bj_HH11_M23 and Kp2Bj_LN294_M23 formed round 1 ± 1 mm translucent plaques on *K. pneumoniae* ST48 lawns (Figures 1A, B, respectively). Kp10Bj_LN54_14 plaques had diameter of 2 ± 1 mm on *K. pneumoniae* (unknown ST) lawns (Figure 1C). Transmission electron microscopy (TEM) analysis of phages particles indicated that Kp1Bj_HH11_M23, Kp2Bj_LN294_M23, and Kp10Bj_LN54_14 phages had an icosahedral heads with a diameter of 86.67 ± 3.15, 93.33 ± 2.55, and 93.33 ± 3.10 nm respectively with long contractile tails length of 126.67 ± 9.55, 126.67 ± 6.10, and 126 ± 10.06 nm respectively (Figures 2A–C).

**FIGURE 1:**
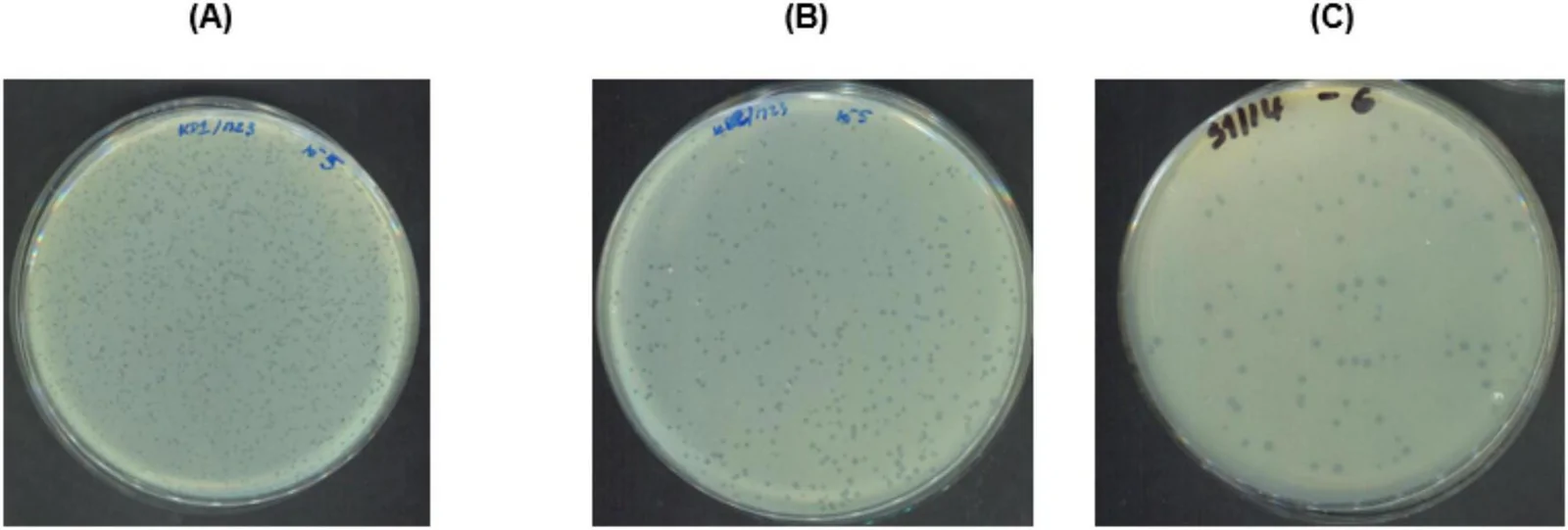
Plaque morphology of three phages formed in a double-layer agar plate with *K. pneumoniae* host strains (Supplementary Table 1). **(A)** Plaque morphology of phage Kp1Bj_HH11_M23. **(B)** Plaque morphology of phage Kp2Bj_LN294_M23. **(C)** Plaque morphology of phage Kp10Bj_LN54_14.

**FIGURE 2:**
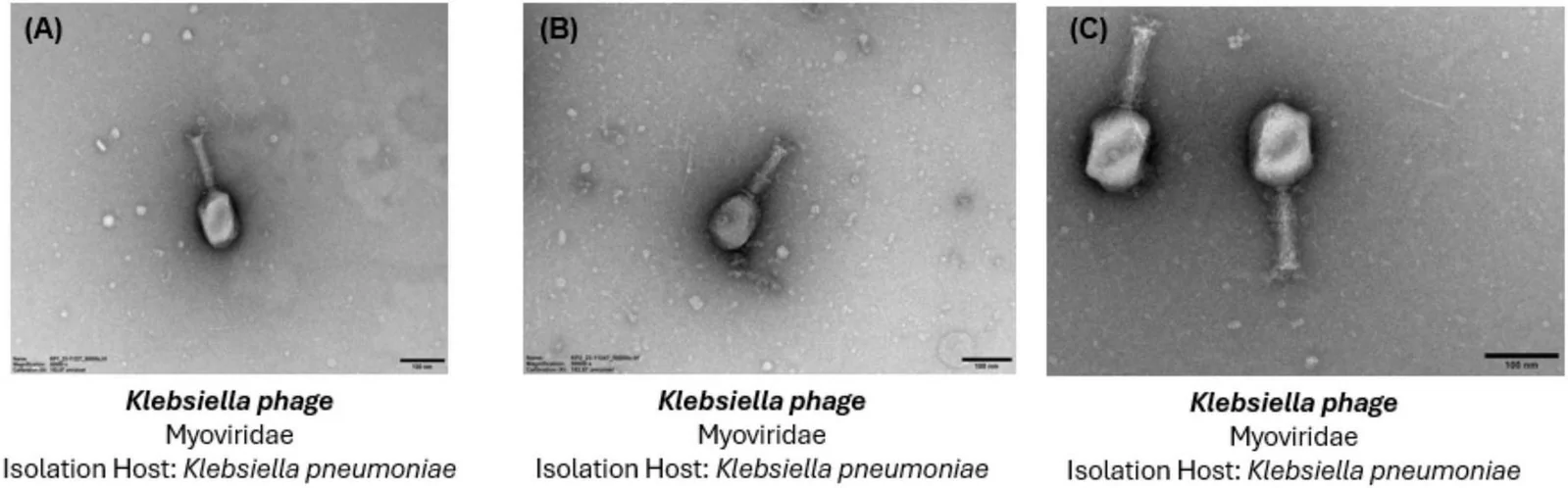
Transmission electron micrographs of phages negatively stained with 1% (w/v) uranyl acetate. The scale bar represents 100 nm. **(A)** Micrograph of phage Kp1Bj_HH11_M23. **(B)** Micrograph of phage Kp2Bj_LN294_M23. **(C)** Micrograph of phage Kp10Bj_LN54_14.

### 3.2 Titer, host range and efficiency of plating (EOP) of isolated phages

Phage titer ranged from 7 × 10^9^ to 2 × 10^10^ PFU/ml. The host range was determined on a panel of 58 *K. pneumoniae* (Supplementary Table 1) and showed that Kp2Bj_LN294_M23 exhibited lytic activity on 74.14% (43 out of 58) of *K. pneumoniae* strains tested with 60.47% of complete lysis plaque and 39.53% of hazy plaques. Kp1Bj_HH11_M23 and Kp10Bj_LN54_14 had lytic activities against 70.69% (41) and 68.97% (40) of *K. pneumoniae* strains tested respectively. Kp1Bj_HH11_M23 had complete lysed plaques on 60.98% strains and 39.02% had hazy lysis. 72.5% of complete lysis plaque and 27.5% of hazy lysis plaque reflects lytic activity of Kp10Bj_LN54_14. Kp2Bj_LN294_M23 phage had the largest percentage of high EOP (7.14%), followed by Kp1Bj_HH11_M23 (5%) (Table 1). The three phages exhibited lytic activity against a wide range of *K. pneumoniae* with a high diversity in capsular loci (KL114, KL53, KL8, KL62, KL64, KL124, KL102, KL57, KL39, KL48, KL17, KL20, KL2, KL30, KL51, KL105, KL146, KL28, KL122, KL38, KL3, KL163).

**TABLE 1:** Host range analysis and EOP of three phages against fifty-eight *K. pneumoniae* strains.

Strains	Phages – Titer (PFU/mL) – EOP^a^		
	Kp1Bj_HH11_M23	Kp2Bj_LN294_M23	Kp10Bj_LN54_14
	7 × 10^9^	2 × 10^10^	7 × 10^9^
KR312			
KR358			
KR431			
KR437			
L02	1E-3	2.86E-5	2.86E-4
L05	2E-2 **	7.5E-6	2.86E-5
L06			
L10			
L11	2.1E^–2^ **	1E-2 *	7.14E-5
L13	7.1E-2 **	6E-4	7.14E-7
L15	4E-4	1E-4	2.86E-5
L19			
L21	2.86E-3	1.5E-3	7.14E-6
L30		2.5E-1 **	2.86E-6
L32			
M31	1.43E-5	1E-4	1.43E-6
M19	1.43E-5	2.5E-4	1.43E-6
M23	–	–	1.43E-4
M20	3.6E-6	1E-3	1.43E-5
M34			8.6E-3
M36	1E-1**	2.5E-3	1.43E-3
M39			1.43E-3
M40	5.7E-2	4E-4	1.43E-3
M41	1.43E-4	1.5E-3	1.43E-3
M54	1.43E-4	6.5E-4	1.43E-3
M61	1.43E-3	6.75E-4	1.43E-3
M63	1.43E-5	3.5E-4	7.14E-4
M71	1.43E-5	1.5E-3	7.14E-6
M45	1.5E-4	2E-3	
M49	7.14E-2	0.5***	0.000714
M57	5.71E-1***	2E-1**	
M13	1.43E-3	3E-4	1.43E-3
M47	2.86E-1**	1E-1**	
M53	2.86E-1**	1E-1**	1.43E-5
M11	1.28E-1^**^	1E-1**	2E-2**
M17	1.43E-4	4.75E-3	1.43E-3
M68	1E-1**	1.3E-3	4.3E-3
M74	2.86E-5	1.1E-5	2.86E-5
M25	1.5E-4	1E-4	7.14E-5
M28	1.43E-4	2E-5	2.86E-1**
M48	5.7E-3	5.E-4	
M32	1E-3	6E-5	5.7E-3
M33			4E-3
M37	1E-1**	5E-1***	2.86E-5
M38	1E-5	3.3E-5	7.14E-6
M42			
M52	1E-1**	5E-2**	1.43E-4
M58	1E-4	1.5E-4	2.86E-6
M65	2.86E-5	1E-5	1.14E-1**
M06	2.86***	4E-1**	7.14E-6
M35		3.5E-1**	
M03	2.9E-1**	50***	4E-2**
M24	4.3E-1**	1E-1**	1E-1**
M69	1E-5	1.43E-5	
M44			
M51	2.86E-6	1.5E-4	1.43E-4
M67			
M75			

Host range: red color = no visible plaque; green color = complete lysis plaque; orange color = hazy lysis plaque.

^a^The numbers in the cells represent the EOP of each phage on the different *K. pneumoniae* strains tested.

***high EOP (EOP ≥ 0.5);

**moderate EOP (0.01<EOP < 0.5);

*low EOP (EOP ≤ 0.01). – no EOP calculation for initial host.

### 3.3 Genomic comparison and phylogenetic analysis

The genomic sequences of Kp1Bj_HH11_M23, Kp2Bj_LN294_M23 and Kp10Bj_LN54_14 are available in the GenBank database under accession numbers PV784030, PV784031 and PV784032, respectively. Kp1Bj_HH11_M23 and Kp2Bj_LN294_M23 are *Marfavirus*, and Kp10Bj_LN54_14 is *Jiaodavirus*. Their genomes have approximately 170 kbp, 40% of GC content, and contained more than 200 viral genes. There were no genes encoding virulence factors or antimicrobial resistance (Table 2). The phylogenetic analysis showed that Kp1Bj_HH11_M23 and Kp2Bj_LN294_M23 are genetically related phages (with more than 99% ANI identity) compared to Kp10Bj_LN54_14. The phylogenetic tree based on the complete genome confirmed that all three phages belong to the Myoviridae family (Figure 3). Kp1Bj_HH11_M23 and Kp2Bj_LN294_M23 genomes encode 122 protein-coding genes (CDS) respectively, and Kp10Bj_LN54_14 encodes 123 CDS. A comparison of the annotated CDSs in the three phage genomes revealed that 186 CDSs in Kp1Bj_HH11_M23, 182 CDSs in Kp2Bj_LN294_M23, and 172 CDSs in Kp10Bj_LN54_14 are predicted to be hypothetical proteins or proteins of unknown function. Genomes contain known proteins related to phage tail, head, and packaging, proteins related to DNA metabolism, and host lysis proteins. Circular maps of the annotated genomes of Kp1Bj_HH11_M23, Kp2Bj_LN294_M23, and Kp10Bj_LN54_14 are shown in Figure 4.

**TABLE 2:** Overview of the phage genomes.

Assembly	Kp1Bj_HH11_M23	Kp2Bj_LN294_M23	Kp10Bj_LN54_14
Accession number	PV784030	PV784031	PV784032
Family	*Myoviridae*	*Myoviridae*	*Myoviridae*
Genus	*Marfavirus*	*Marfavirus*	*Jiaodavirus*
Completeness	99.34	99.34	99.07
Contamination	0.0	0.0	0.0
CDS	308	304	295
Number of contigs	1	1	1
Largest contig	170568	170567	167615
Total length	170568	170567	167615
GC (%)	40.69	40.71	39.53
N50	170568	170567	167615
Antibiotic resistance genes	None	None	None
Identity to Kp1Bj_HH11_M23	100%	99.69%	81.44%
Identity to Kp2Bj_LN294_M23	99.70%	100%	80.31%
Identity to Kp10Bj_LN54_14	82.74%	83.24%	100%

**FIGURE 3:**
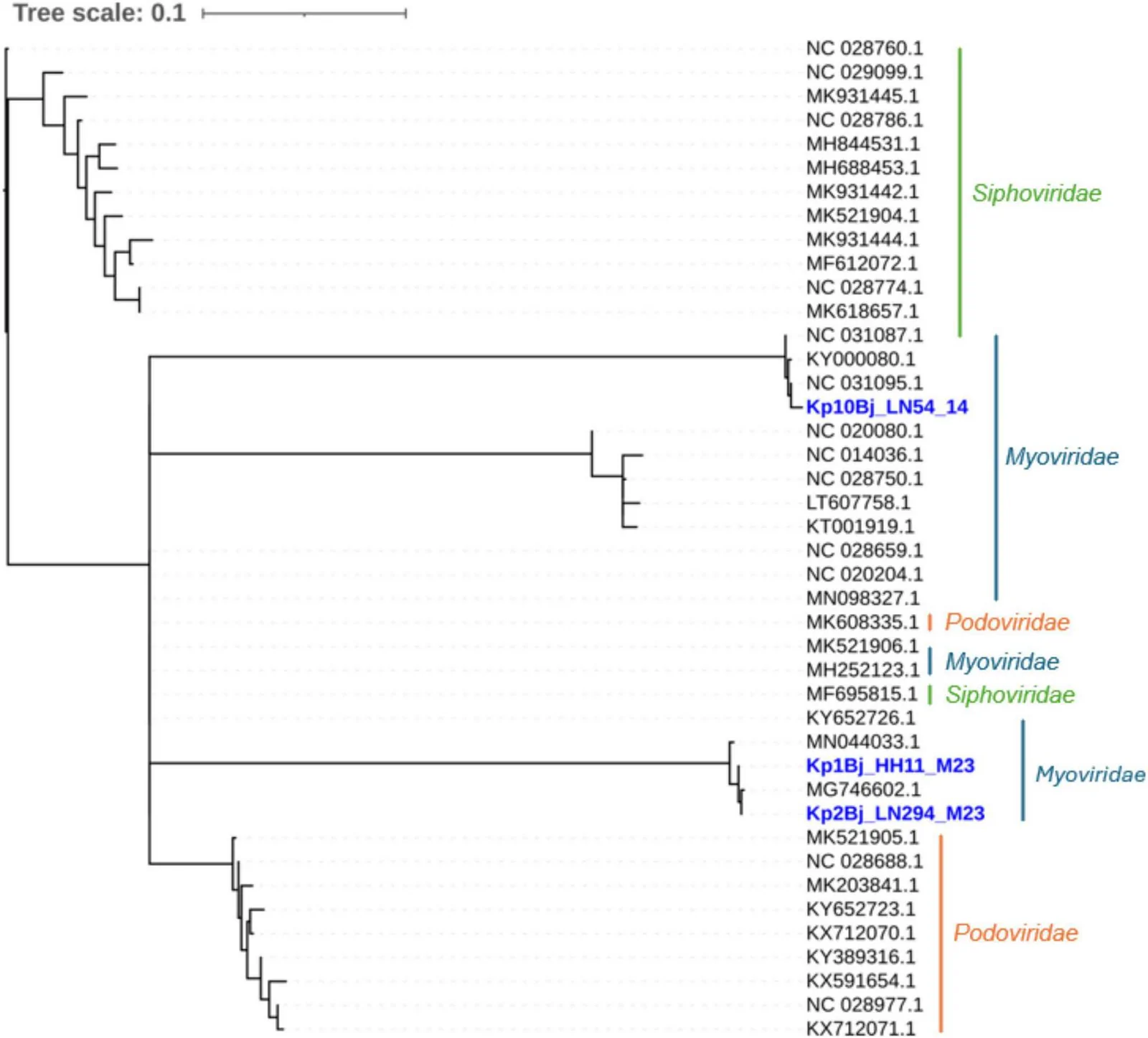
Phylogenetic tree of 42 phage genomes based on accessory genome content. *Klebsiella pneumoniae* reference phages are highlighted in blue.

**FIGURE 4:**
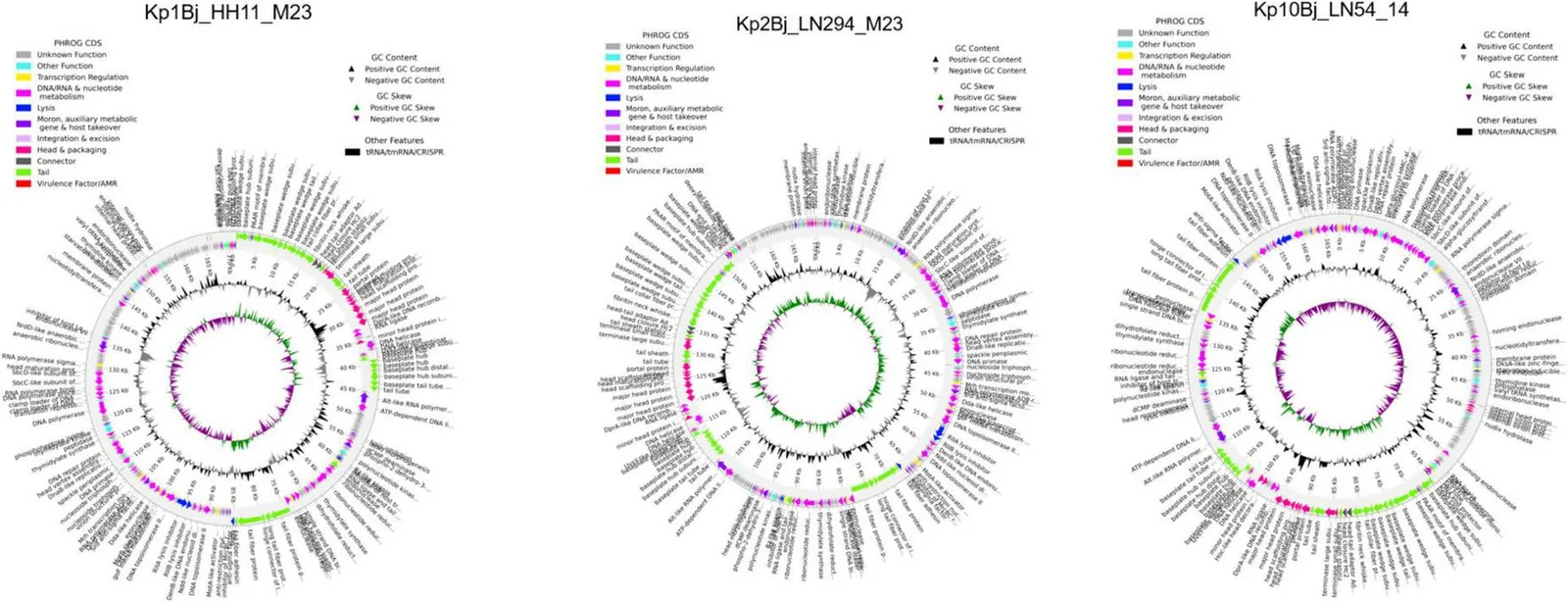
Representative genome content annotation of phages.

Nine pairwise comparisons used for the BLASTn analysis were examined: Kp1Bj_HH11_M23, and Kp2Bj_LN294_M23 were each compared with vB_Kpn_F48 (MG746602), Marfa phage (MN044033) and vB_KpnP_Henu1_3 (PQ133004), while Kp10Bj_LN54_14 was compared with KPV15 (KY000080), vB_KpnM_KpV477 (NC_031087) and PK0111 (NC_031095). Whole-genome comparisons demonstrated extensive nucleotide similarity between Kp1Bj_HH11_M23/Kp2Bj_LN294_M23 and two of their selected homologues (Table 3). Kp1Bj_HH11_M23, and Kp2Bj_LN294_M23 each showed approximately 96.9% nucleotide identity with vB_Kpn_F48, with 96.9% of the Kp1Bj_HH11_M23/Kp2Bj_LN294_M23 genomes and 96.8% of the vB_Kpn_F48 genome represented by homologous regions. Both phages also showed approximately 92.5% nucleotide identity with Marfa phage, covering 92.7% of the Kp1Bj_HH11_M23/Kp2Bj_LN294_M23 genomes and approximately 93.8% of Marfa phage. A markedly different result was obtained for vB_KpnP_Henu1_3. This phage has a substantially smaller genome (49,808 bp) than Kp1Bj_HH11_M23 (170,568 bp) and Kp2Bj_LN294_M23 (170,567 bp). No significant BLASTn nucleotide alignment was detected between vB_KpnP_Henu1_3 and either Kp1Bj_HH11_M23 or Kp2Bj_LN294_M23, including when the comparisons were repeated using BLASTn with an E-value threshold of 1 × 10^–3^. Thus, a nucleotide similarity percentage could not be meaningfully calculated for these comparisons, rather than the similarity being interpreted as 0%. Kp10Bj_LN54_14 showed extensive nucleotide similarity with all three selected homologues: 95.2% identity with KPV15 (94.1% query coverage and 94.4% reference coverage), 94.8% with vB_KpnM_KpV477 (92.3% and 91.9% coverage, respectively), and 94.4% with PK0111 (93.1% and 92.5% coverage, respectively). Fragment representation was high for all seven comparisons for which ANI could be calculated, with 52–55 matched fragments out of 55–56 total fragments (Table 3). The Proksee whole-genome comparisons supported the relationships identified by BLASTn and FastANI (Figure 5). Kp1Bj_HH11_M23 and Kp2Bj_LN294_M23 showed extensive, nearly genome-wide conservation with vB_Kpn_F48 (MG746602) and Marfa phage (MN044033), with the comparative tracks spanning most of the respective reference genomes and showing only localized interruptions or regions of reduced similarity (Figures 5A, B). In contrast, no substantial homologous track was detected for vB_KpnP_Henu1_3 (PQ133004) against either Kp1Bj_HH11_M23 or Kp2Bj_LN294_M23, consistent with the absence of significant BLASTn alignments and the lack of a FastANI estimate for these comparisons. Kp10Bj_LN54_14 showed similarly extensive genome-wide conservation with all three selected homologues, KPV15 (KY000080), vB_KpnM_KpV477 (NC_031087) and PK0111 (NC_031095), while several localized regions of reduced or absent similarity remained apparent (Figure 5C).

**TABLE 3:** Comparison of three studied phages and homologous phages.

Query	Reference	Query length (bp)	Reference length (bp)	Query coverage (%)	Weighted identity (%)	Reference coverage (%)	Matched fragments	Total fragments
Kp1Bj_HH11_M23	vB_Kpn_F48	170568	170764	96.88	96.89	96.81	55	56
Kp1Bj_HH11_M23	Phage Marfa	170568	168532	92.65	92.52	93.83	54	56
Kp1Bj_HH11_M23	vB_KpnP_Henu1_3	170568	49808	0	NA	0	0	NA
Kp2Bj_LN294_M23	vB_Kpn_F48	170567	170764	96.88	96.85	96.81	54	56
Kp2Bj_LN294_M23	Phage Marfa	170567	168532	92.65	92.54	93.82	52	56
Kp2Bj_LN294_M23	vB_KpnP_Henu1_3	170567	49808	0	NA	0	0	NA
Kp10Bj_LN54_14	KPV15	167615	167034	94.08	95.22	94.39	54	55
Kp10Bj_LN54_14	vB_KpnM_KpV477	167615	168272	92.32	94.76	91.85	52	55
Kp10Bj_LN54_14	PK0111	167615	168758	93.12	94.35	92.46	53	55

**FIGURE 5:**
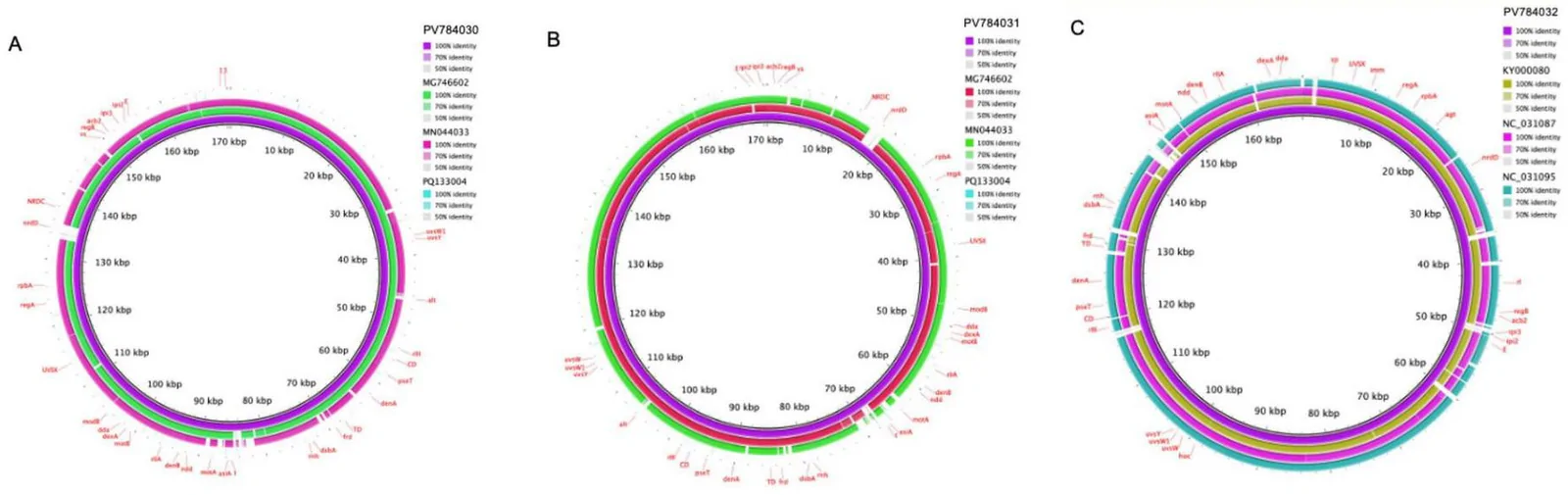
Compared genomic analysis of the three studied phages. **(A)** Alignment of Kp1Bj_HH11_M23 (PV784030) with genomes of other phages (vB_Kpn_F48, MG746602; phage Marfa, MN044033; vB_KpnP_Henu1_3, PQ133004) infected hypervirulent *K. pneumoniae* strains. **(B)** Alignment of Kp2Bj_LN294_M23 (PV784031) with genomes of other phages (vB_Kpn_F48, MG746602; phage Marfa, MN044033; vB_KpnP_Henu1_3, PQ133004) infected hypervirulent *K. pneumoniae* strains. **(C)** Alignment of Kp10Bj_LN54_14 (PV784032) with genomes of other phages (KPV15, KY000080; vB_KpnM_KpV477, NC031072; PK0111, NC031095) infected hypervirulent *K. pneumoniae* strains.

### 3.4 Characteristics of phages growth

Phage Kp1Bj_HH11_M23 had a higher titer at multiplicity of infection (MOI) 10, which was different from the titers obtained at the other MOIs. This difference was not significant at MOI 100, it was highly significant at MOIs 0.001, 0.01, 0.1 and 1 (Figure 6A). The highest titers of phages Kp2Bj_LN294_M23 and Kp10Bj_LN54_14 were obtained at MOIs 0.1 and 0.001 respectively. The difference between these titers and those obtained at the other MOIs (0.01, 1, 10, 100) was not significant (Figures 6B, C). The different kinetic adsorption curves of phages at their optimal MOI showed that Kp1Bj_HH11_M23 and Kp2Bj_LN294_M23 were completely absorbed at 30 min. Kp10Bj_LN54_14 was not completely adsorbed at 60 min (Figure 7). One-step growth curve analysis of phages showed that both phages Kp1Bj_HH11_M23 and Kp2Bj_LN294_M23 have a latency time of 10 min, while that of phage Kp10Bj_LN54_14 is 5 min. In addition, the burst sizes of phages Kp1Bj_HH11_M23, Kp2Bj_LN294_M23, and Kp10Bj_LN54_14 were 402, 2208, and 119 PFU/cell, respectively (Figure 8).

**FIGURE 6:**
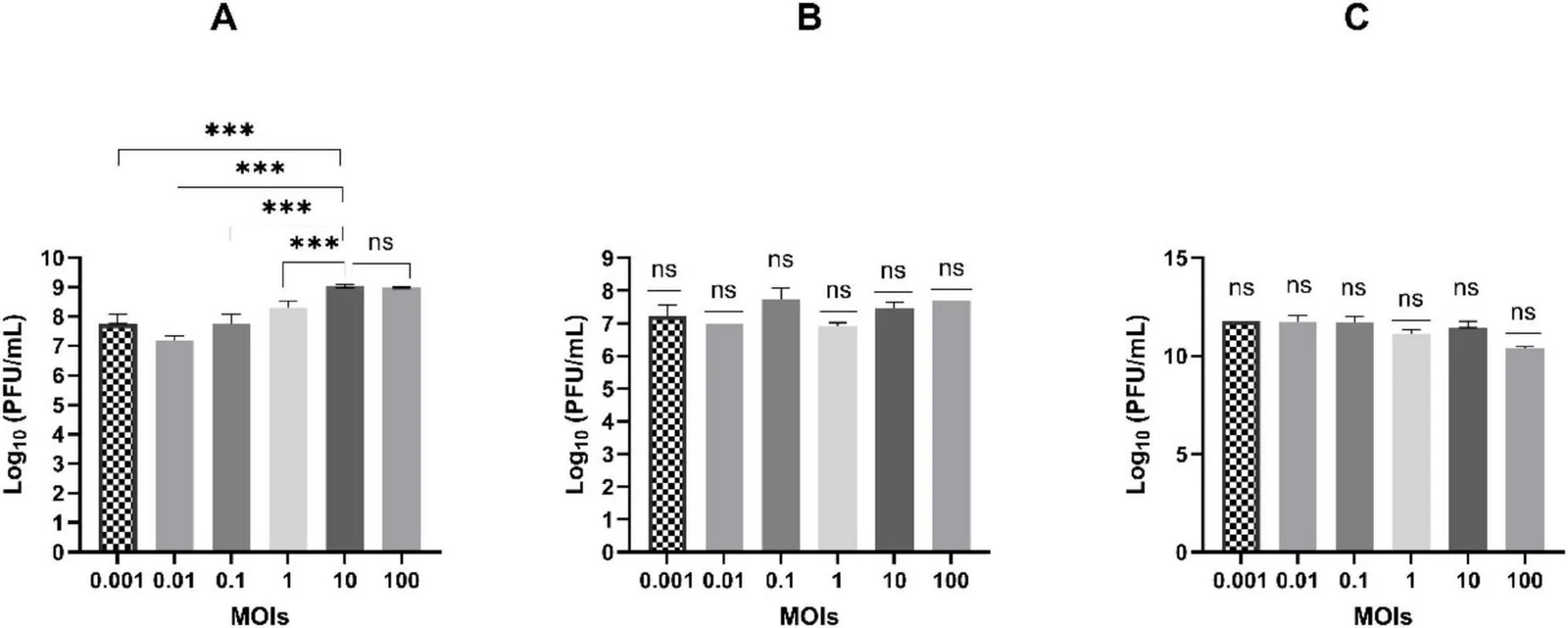
Titer of each phage at different MOIs. **(A)** Titer of Kp1Bj_HH11_M23 at different MOIs. **(B)** Titer of Kp2Bj_LN294_M23 at different MOIs. **(C)** Titer of Kp10Bj_LN54_14 at different MOIs. The data are presented as the mean ± standard deviation of three independent experiments. The asterisks indicate significant differences between the MOIs (***P < 0.001, Ordinary one-way ANOVA followed by Tukey’s multiple comparisons test).

**FIGURE 7:**
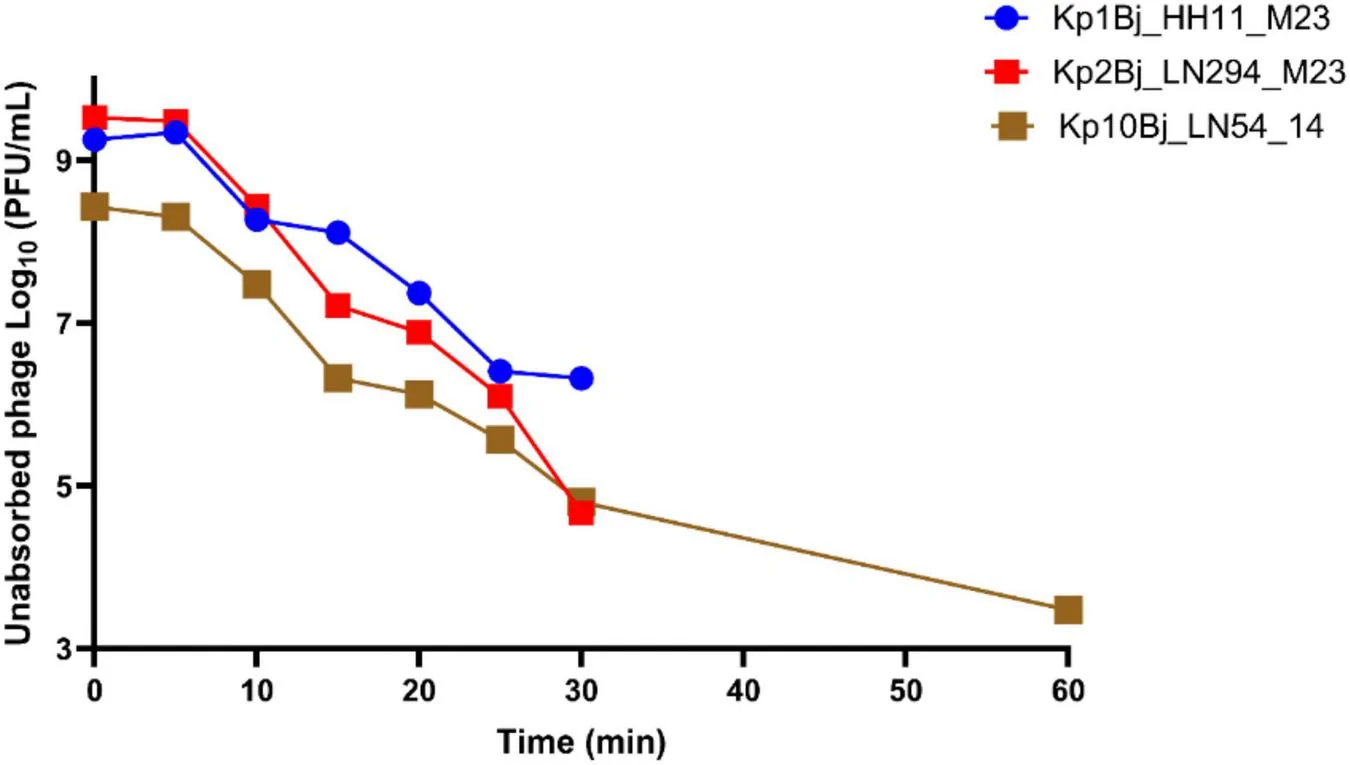
Kinetic adsorption of phages to the host strains surfaces at their optimal MOI.

**FIGURE 8:**
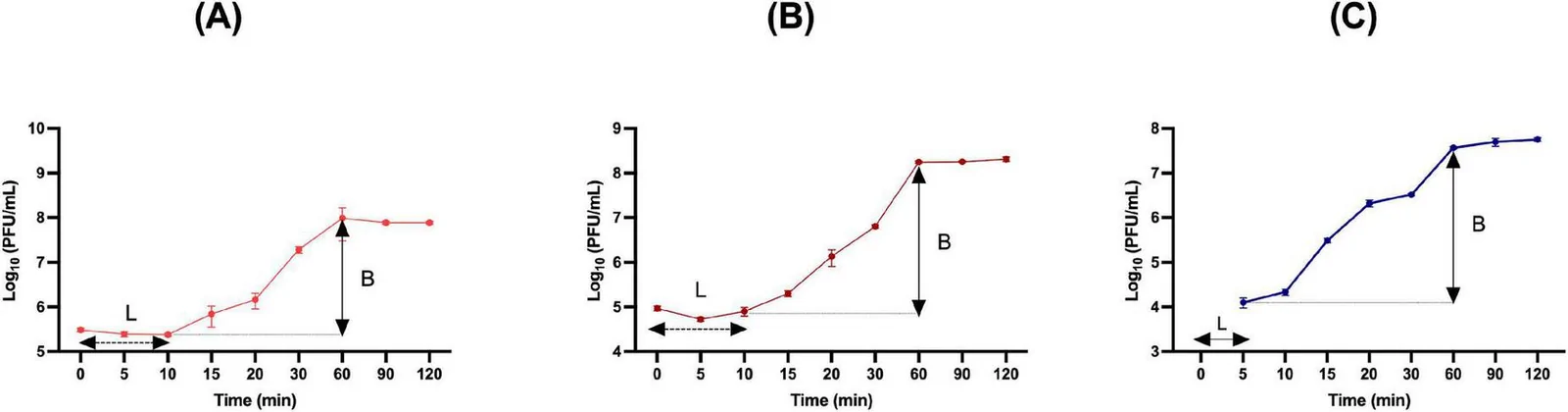
One-step growth curve analysis of phages at their optimal MOI. **(A)** One-step growth curve of Kp1Bj_HH11_M23. **(B)** One-step growth curve of Kp2Bj_LN294_M23. **(C)** One-step growth curve of Kp10Bj_LN54_14.

### 3.5 Antibiofilm activities

The phage-treated groups showed a reduction in the planktonic cells compared with the control group after both 24 h and 48 h of incubation. This decrease of planktonic cells by Kp1Bj_HH11_M23 was highly significant at 48 h and not significant at 24 h. Reduction of planktonic cells by Kp2Bj_LN294_M23 was significant in 24 h and very significant in 48 h. While the reduction of planktonic cells by phage Kp10Bj_LN54_14 was highly significant at 24 h (Figures 9A–C). Phages showed activity in destroying the biofilms formed within 24 and 48 h. A decrease in the OD_600nm_ of biofilms in the phage-treated groups compared with the control groups was observed. However, the destructive effect of Kp1Bj_HH11_M23 and Kp10Bj_LN54_14 on the biofilms formed in 24 h was not significant and was very significant in 48 h compared to the control (Figures 10A–C). In contrast, the destruction of biofilm by Kp2Bj_LN294_M23 was highly significant at 24 h and not significant at 48 h compared to the control (Figure 10B). In more quantitative terms, the three phages destroyed more than 65% of the biofilms formed by the *K. pneumoniae* strains used. Regarding the multidrug resistant and hypervirulent *K. pneumoniae* ST48 (KL124) strain, Kp1Bj_HH11_M23 exhibited strong antibiofilm activity compared to Kp2Bj_LN294_M23, with biofilm disruption rates of 91.15 % (control: 2.6 × 10^9^ ± 1.5 × 10^9^ CFU/mL, phage treated: 2.3 × 10^8^ ± 7.2 × 10^7^ CFU/mL, 95% CI 4.8 × 10^9^ – 1.8 × 10^7^) and 98.46 % (control: 1.3 × 10^10^ ± 5.8 × 10^9^ CFU/mL, phage treated: 2 × 10^8^ ± 1 × 10^8^ CFU/mL, 95% CI 2.2 × 10^10^ – 3.9 × 10^9^), respectively, at 24 and 48 h. As for Kp10Bj_LN54_14, it exhibited significant activity, achieving nearly total disruption of biofilm formed by clinical strain 14 within 48 h (99.96%) (control: 7.7 × 10^10^ ± 1.5 × 10^8^ CFU/mL, phage treated: 2.7 × 10^7^ ± 2.1 × 10^7^ CFU/mL, 95% CI 1.3 × 10^9^ – 7.9 × 10^8^) (Table 4).

**FIGURE 9:**
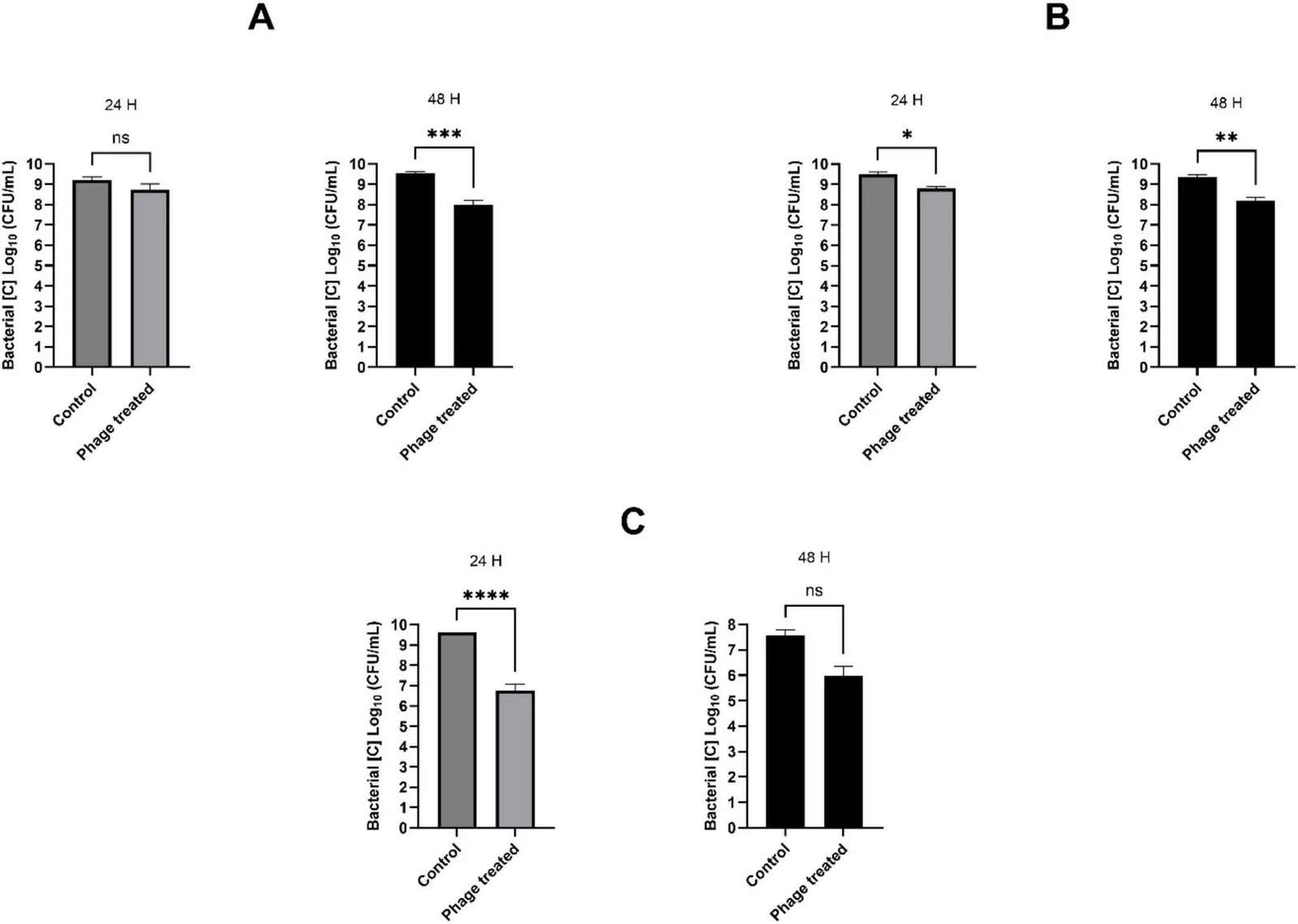
Impact of phage treatment on host cell viability, for 24 to 48 h before counting viable bacterial cells (CFU/ml). **(A)** Impact of Kp1Bj_HH11_M23 treatment on host cell viability. **(B)** Impact of Kp2Bj_LN294_M23 treatment on host cell viability. **(C)** Impact of Kp10Bj_LN54_14 treatment on host cell viability. The data are presented as the mean ± standard deviation of three independent experiments. The asterisks indicate significant differences between control and phage treated groups (*P < 0.05, **P < 0.01, ***P < 0.001 or ****P < 0.0001, unpaired *t*-test).

**FIGURE 10:**
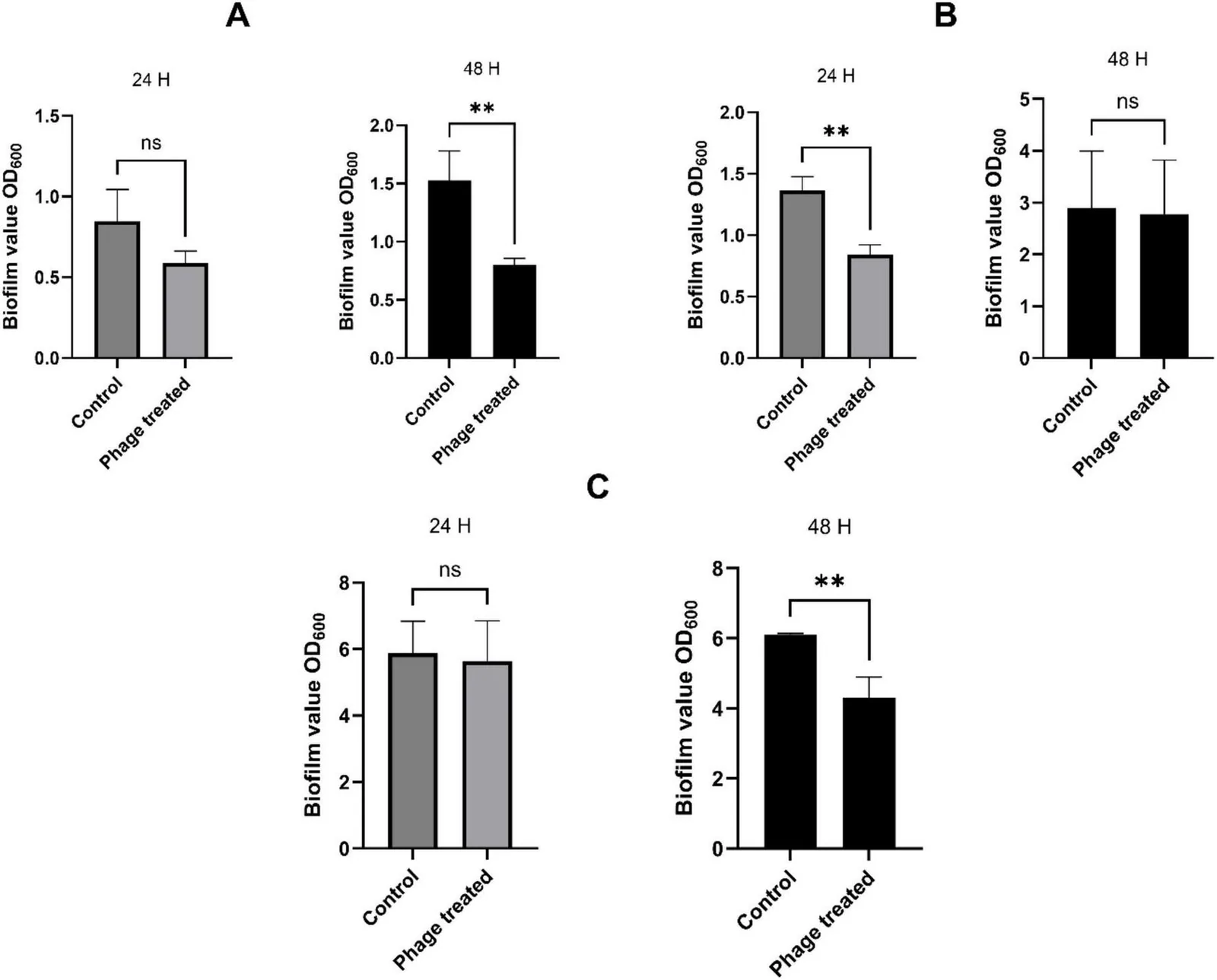
Antibiofilm activities of phages against host cells in LB medium. OD_600_ was measured after 24 and 48 h. **(A)** Antibiofilm activity of Kp1Bj_HH11_M23. **(B)** Antibiofilm activity of Kp2Bj_LN294_M23. **(C)** Antibiofilm activity of Kp10Bj_LN54_14. The data are presented as the mean ± standard deviation of three independent experiments. The asterisks indicate significant differences between control and phage treated groups (**P < 0.01, unpaired *t*-test).

**TABLE 4:** Quantitative effect of phages on biofilm disruption compared to the control.

Phages	Concentration biofilm (CFU/mL), percentage of biofilm disruption (BD) and *p*-value									
	24 h					48 h				
	Control	Phage treated	95% CI	BD (%)	*p*-value	Control	Phage treated	95% CI	BD (%)	*p*-value
Kp1Bj_HH11_M23	2.6 × 10^9^ ± 1.5 × 10^9^	2.3 × 10^8^ ± 7.2 × 10^7^	4.8 × 10^9^ − 1.8 × 10^7^	91.15	0.0511	1.3 × 10^10^ ± 5.8 × 10^9^	2 × 10^8^ ± 1 × 10^8^	2.2 × 10^10^ − 3.9 × 10^9^	98.46	0.0170
Kp2Bj_LN294_M23	3 × 10^9^ ± 0	1 × 10^9^ ± 2 × 10^8^	2.3 × 10^9^ − 1.6 × 10^9^	65.56	<0.0001	2 × 10^9^ ± 1 × 10^9^	4 × 10^8^ ± 2 × 10^8^	3.2 × 10^9^ − 3.4 × 10^7^	80	0.0531
Kp10Bj_LN54_14	1.7 × 10^9^ ± 1.2 × 10^9^	2.8 × 10^8^ ± 7 × 10^7^	3.2 × 10^9^ − 4.7 × 10^8^	83.53	0.1065	7.7 × 10^10^ ± 1.5 × 10^8^	2.7 × 10^7^ ± 2.1 × 10^7^	1.3 × 10^9^ − 7.9 × 10^8^	99.96	0.003

### 3.6 Stability

Phages were sensitive to pH 1 and 2, losing their lytic activity on their respective hosts in an extremely acidic environment (Figures 11A–C). Unlike Kp1Bj_HH11_M23 and Kp2Bj_LN294_M23, Kp10Bj__LN54_14 was sensitive in pH 12, 13 and 14, and therefore inactive in an extremely basic environment (Figure 11C). Phages were stable in conditions with a pH between 3 and 11, inclusive, with titers from of 10^7^ to 10^9^, like their initial titers. Above 70 °C, phages showed no lytic activity on their respective hosts. Kp1Bj_HH11_M23 and Kp2Bj_LN294_M23 were stable over the temperature range from 4 to 60 °C with a significant decrease in titers at 70 °C (Figures 12A, B). Kp10Bj_LN54_14 was stable from 4 to 50 °C and showed a significant decrease in titer at 60 and 70 °C (Figure 12C).

**FIGURE 11:**
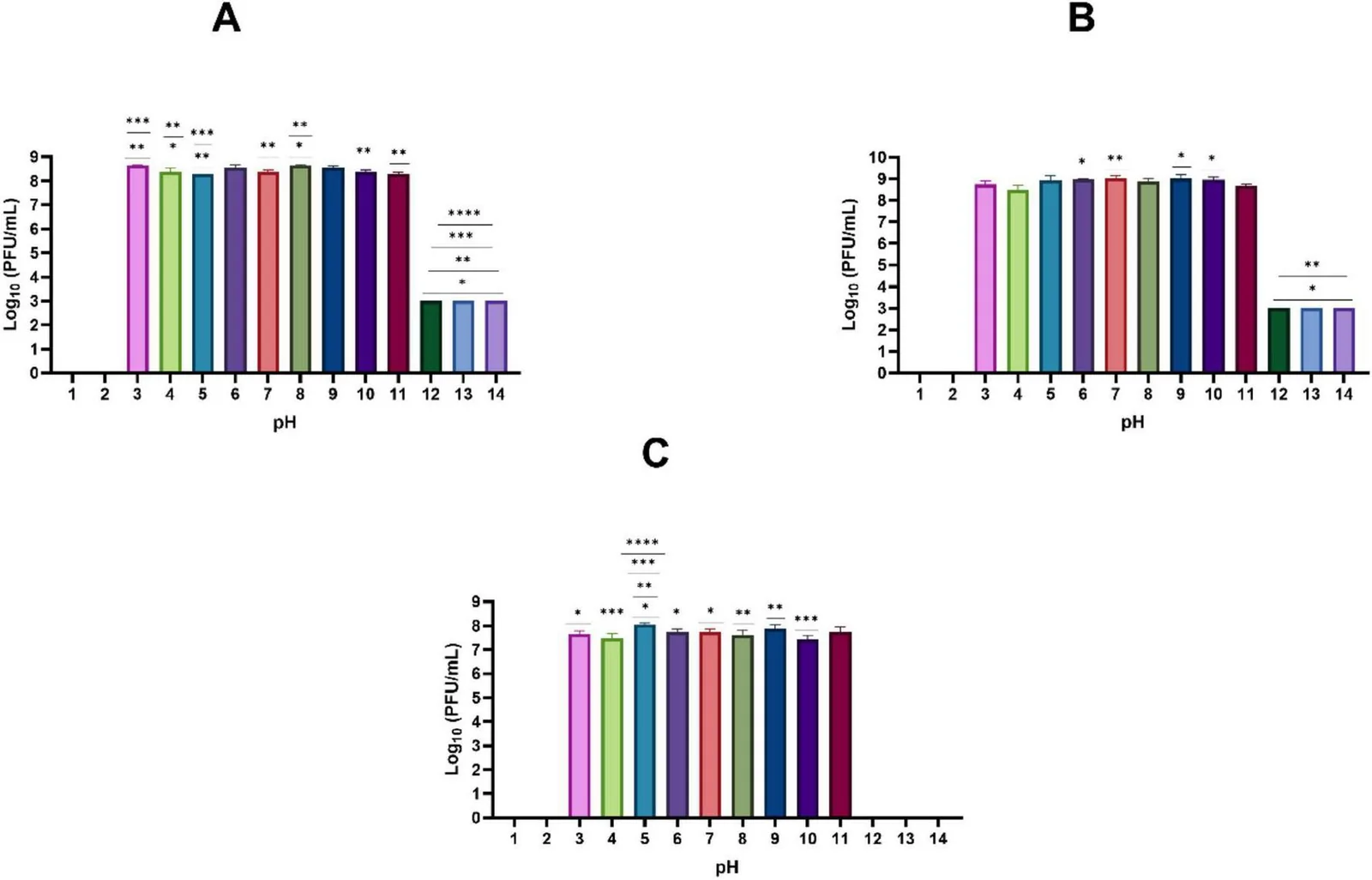
Stability of phages at different pH conditions (1 to 14). **(A)** Kp1Bj_HH11_M23 stability. **(B)** Kp2Bj_LN294_M23 stability. **(C)** Kp10Bj_LN54_14 stability. The data are presented as the mean ± standard deviation of three independent experiments. The asterisks indicate significant differences between the pH conditions (*P < 0.05, **P < 0.01, ***P < 0.001 or ****P < 0.0001, Ordinary one-way ANOVA followed by Tukey’s multiple comparisons test).

**FIGURE 12:**
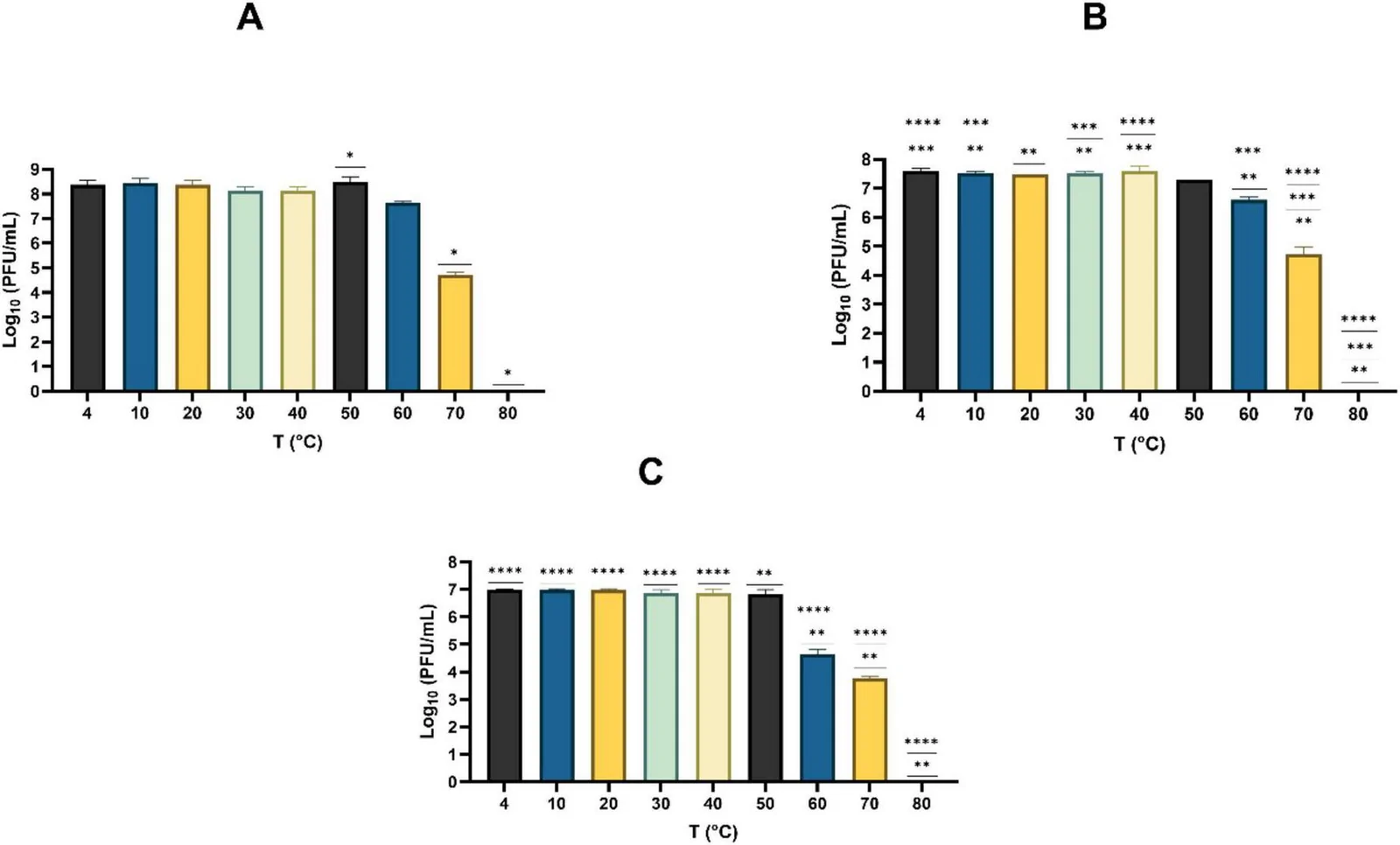
Stability of phages at different temperatures. **(A)** Kp1Bj_HH11_M23 stability. **(B)** Kp2Bj_LN294_M23 stability. **(C)** Kp10Bj_LN54_14 stability. The data are presented as the mean ± standard deviation of three independent experiments. The asterisks indicate significant differences between temperature conditions (*P < 0.05, **P < 0.01, ***P < 0.001 or ****P < 0.0001, Ordinary one-way ANOVA followed by Tukey’s multiple comparisons test).

## 4 Discussion

The global increase in antimicrobial resistance (AMR) poses a significant challenge, particularly for low- and middle-income countries (LMICs), where roughly 90% of the worldwide deaths from AMR are expected to occur (Murray et al., 2022). This problem has led to the emergence of phage therapy as a promising alternative to antibiotics, whether used alone, in phage cocktails, or in combination with antibiotics. Lytic phages are considered one of the best solutions for treating infections caused by multidrug-resistant bacteria (Bleriot et al., 2023). Currently, most research on phage therapy is concentrated in high-income countries (Eskenazi et al., 2022; Lebeaux et al., 2021; Jennes et al., 2017; Leitner et al., 2017; Międzybrodzki et al., 2012). There is no data available on the effectiveness of phage therapy in Africa, where bacterial epidemiology and healthcare infrastructure differ substantially from those in high-income countries. Furthermore, it is crucial to generate data on lytic phages that are candidates for therapeutic use in the treatment of infections caused by multidrug resistant bacteria in Africa. The present study aims to evaluate the potential contributions of phages in reducing bacterial growth and degrading biofilms formed by multidrug resistant and hypervirulent *Klebsiella pneumoniae* strains. After characterizing the isolated phages in Benin, their antibacterial activity was evaluated *in vitro* in a biofilm model.

*Klebsiella pneumoniae* exhibits high resistance to a broad spectrum of drugs, including beta-lactams, fluoroquinolones and aminoglycosides, and is responsible for many human infections (Mulani et al., 2022). In this context, the use of specific and personalized phage cocktails may be essential in the fight against multidrug resistant bacteria of the genus *Klebsiella* (Das and Kaledhonkar, 2024). For this, the constant isolation of novel *Klebsiella* phages capable of killing antibiotic-resistant *Klebsiella* and a thorough understanding of the biology of these phages are essential to develop new antibacterial strategies that could be used to treat human pathogens (Fayez et al., 2023). As part of this study, three novel Myoviruses were isolated in Benin from samples of hospital effluent (Kp1Bj_HH11_M23), stool (Kp10Bj_LN54_14), and animal feces (Kp2Bj_LN294_M23), respectively. Which shows that lytic phages can be found in human, animal and environmental samples. The three phages showed lytic activity on a wide range of clinical strains of *K. pneumoniae* (at least 40 strains) with various sequence types. Importantly, they can infect clinical isolates with different capsular loci, including KL114, KL53, KL8, KL62, KL64, KL124, KL102, KL57, KL39, KL48, KL17, KL20, KL2, KL30, KL51, KL105, KL146, KL28, KL122, KL38, KL3, and KL163. The diversity of *Klebsiella* capsules is the main obstacle to the development of broad-spectrum phage cocktails. Since many phages use specific capsular polysaccharides (CPS) as receptors, a single phage often infects only one or a few of the many known capsular serotypes of *Klebsiella*, which limits its clinical efficacy (Chen et al., 2024; Ferriol-González et al., 2024; Wang et al., 2024; Burke et al., 2025). These results show that the three phages could be a good candidate for development of broad-spectrum phage cocktails and therapy against infections (urinary tract infections, pneumonia, sepsis, nosocomial infections) caused by hypervirulent and antibiotic-resistant (even last resort) *K. pneumoniae* strains. With a wide host range (from 68.97% to 74.14%), the three *Klebsiella* phages have a broader lytic spectrum than some previously characterized novel *Klebsiella* phages (Ciacci et al., 2018; Bleriot et al., 2023; Fang et al., 2023). Phage vB_Kpn_F48 has a host range of 22.95% (14/61) among hypervirulent strains of *K. pneumoniae* of the same capsular serotype, KL17 (Ciacci et al., 2018). Phages vB_KpnS_VAC35 and vB_KpnM_VAC36 infected 36.17% (17/47) and 38.29% (18/47), respectively, of the multidrug resistant and hypervirulent *K. pneumoniae* strains with a variety of capsular serotypes (KL30, KL15, KL51, KL52, KL64, KL126, KL24, KL36, KL38…) (Bleriot et al., 2023). Of 33 multidrug resistant and hypervirulent *K. pneumoniae* strains, each phage vB_KpnS_SCNJ1 and vB_KpnM_SCNJ1-Y infected only one strain (ST29, KL54), representing an infection rate of 3.03% (Fang et al., 2023). The capsular serotypes of *K. pneumoniae* strains are key determinants of phage infection (Haudiquet et al., 2024). *Klebsiella* phages have developed mechanisms that allow them to use the capsule as a receptor or simply to degrade it by producing depolymerase (Pires et al., 2016). These phages can recognize and bind to the capsules for which they encode a depolymerase. If the phage possesses a specific depolymerase to the strain’s capsular type, it will degrade the capsule, adsorb effectively, and initiate a complete lytic cycle, resulting in a high EOP. Otherwise, it will be unable to penetrate the capsule. Adsorption fails, leading to a low or zero EOP. Phages Kp1Bj_HH11_M23 and Kp2Bj_LN294_M23 have high EOP values for hosts of different capsular serotypes (KL17, KL28, KL38, KL48, KL122, and KL124), which would suggest that these two phages possess depolymerases for multiple capsular serotypes of *K. pneumoniae*. Phage Kp10Bj_LN54_14 exhibits moderate EOPs for certain strains of the same capsular serotype (KL38 and KL122) for which phages Kp1Bj_HH11_M23 and Kp2Bj_LN294_M23 have high EOPs. These results could be explained by the fact that, despite producing a depolymerase to degrade these capsules, the biological characteristics of phage Kp10Bj_LN54_14 (adsorption time: 60 min, burst size: 119 PFU/cell) did not allow it to achieve high EOPs like those of phages Kp1Bj_HH11_M23 (adsorption time: 30 min, burst size: 402 PFU/cell) and Kp2Bj_LN294_M23 (adsorption time: 30 min, burst size: 2,208 PFU/cell).

The morphology of the three phages (Kp1Bj_HH11_M23, Kp2Bj_LN294_M23 and Kp10Bj_LN54_14) was observed using a transmission electron microscope and indicated that they had Myovirus, and all belonged to the order Caudovirales. This result was confirmed by phylogeny, which showed the proximity of the three phages to other phages in the same family (Myoviridae). This classification corresponds to that of other phages known to infect clinical *K. pneumoniae* (Min et al., 2019; Zurabov and Zhilenkov, 2019; Fang and Zong, 2022; Fang et al., 2023; Gorodnichev et al., 2025; Gou et al., 2025). Furthermore, phages Kp1Bj_HH11_M23 and Kp2Bj_LN294_M23 belong to the *Marfavirus* genus, while Kp10Bj_LN54_14 belongs to the *Jiaodavirus* genus. Phylogeny has shown that phages Kp1Bj_HH11_M23 and Kp2Bj_LN294_M23 are closely related to phages Marfa (MN044033) and vB_Kpn_F48 (MG746602), which are also phages belonging to the genus *Marfavirus* (Ciacci et al., 2018; Harb et al., 2019). Similarly, the phylogenetic tree showed that Kp10Bj_LN54_14 is closely related to the phages PK0111 (NC_031095) and KPV15 (KY000080). PK0111 is a phage capable of infecting clinical strains of *K. oxytoca* and *K. pneumoniae* (Park et al., 2017). KPV15 is a phage that is part of the phage cocktail used for the treatment and prophylaxis of healthcare-associated infections in a mouse model and in phage therapy in patients with infections related to cardiothoracic surgery (Aleshkin et al., 2016; Rubalskii et al., 2020). Thus, Kp10Bj_LN54_14 could be a candidate for phage therapy against healthcare-associated infections caused by bacteria of the genus *Klebsiella*. A comparison of the genomes of the three phages (Kp1Bj_HH11_M23, Kp2Bj_LN294_M23 and Kp10Bj_LN54_14) with their homologues (vB_Kpn_F48, Marfa phage, KPV15, vB_KpnM_KpV477 and PK0111), reveals conservation at the genome-wide level, with significant localized differences within the genomes. This explains the differences and the novel nature of phages Kp1Bj_HH11_M23, Kp2Bj_LN294_M23 and Kp10Bj_LN54_14 compared to previous *Klebsiella* phages. Genome analysis showed that there are no genes associated with recombinases, integrases, or repressors in any of the three phage genomes, confirming that all three phages follow a lytic life cycle. The absence of genes linked to bacterial virulence or antibiotic resistance in each of the three phage genomes indicates that these new lytic phages meet the generally accepted genomic criteria for therapeutic phages (Al-Anany et al., 2021; Song et al., 2021).

According to the one step growth curve, Kp2Bj_LN294_M23 has the largest burst size (2208 PFU/cell), followed by Kp1Bj_HH11_M23 (402 PFU/cell) and Kp10Bj_LN54_14 (119 PFU/cell). Likewise, Kp2Bj_LN294 is larger than previously reported *Klebsiella* phages vB_Kpn_ZC2 (650 PFU/cell), vB_KpnP_kP17 (331 PFU/cell), vB_KleS-HSE3 (277 PFU/cell) and IME183 (135 PFU/cell) (Xu et al., 2023; Abdel-Razek et al., 2025; Fayez et al., 2023; Peng et al., 2020). The rapid phage adsorption and massive release of virions suggest that Kp1Bj_HH11_M23, Kp2Bj_LN294_M23 and Kp10Bj_LN54_14 might have robust therapeutic efficacy. The optimal multiplicity of infections (MOIs) of the three phages (Kp1Bj_HH11_M23, Kp2Bj_LN294_M23 and Kp10Bj_LN54_14) are 10, 0.1, and 0.001, respectively, where they produce the highest phage titers. This difference in optimal MOI among the three phages is due to their differing biological characteristics. Phage Kp10Bj_LN54_14 has a shorter latency period (5 min) than the other two. Therefore, it replicates very rapidly inside the host cell. This, combined with its burst size of 119 PFU/cell and adsorption time (60 min), explains its very low MOI (0.001). Phages Kp1Bj_HH11_M23 and Kp2Bj_LN294_M23 have the same latency period, same adsorption time (30 min) and the same host. However, their burst sizes differ. Phage Kp2Bj_LN294_M23 (2,208 PFU/cell) has a burst size 5.5 times that of phage Kp1Bj_HH11_M23 (402 PFU/cell). Thus, with a more optimal amplification capacity, just a few waves of infection with Kp2Bj_LN294_M23 are sufficient to saturate the medium with viral particles. This explains the low MOI (0.1) of Kp2Bj_LN294_M23 compared to Kp1Bj_HH11_M23 (MOI = 10). According to Abedon (1989) and Chen et al. (2023), producing large quantities of phages using a lower MOI and a shorter incubation period may prove to be less expensive. Thus, phages Kp10Bj_LN54_14 and Kp2Bj_LN294_M23 may be considerably better than Kp1Bj_HH11_M23 in terms of optimal phage production.

The time-killing assay enabled us to determine phage virulence against *K. pneumoniae* planktonic cells. The growth of planktonic cells was significantly inhibited by the three phages within 48 h of incubation. Phages Kp1Bj_HH11_M23, Kp2Bj_LN294_M23 and Kp10Bj_LN54_14 demonstrated a significant ability to degrade *K. pneumoniae* (ST48 - KL124; and classical strain 14) preformed biofilms *in vitro*. At the same MOI, phage Kp2Bj_LN294_M23 exhibits relatively lower antibiofilm activity than phage vB_Kp_H122 against a multidrug resistant and hypervirulent *K. pneumoniae* KL57 strain such as the one used in this study (Fan et al., 2026). The same observation was made for phage Kp10Bj_LN54_14, which exhibits lower antibiofilm activity than phage vB_KpnP_Henu1_3. In contrast, phages Kp1Bj_HH11_M23 and Kp2Bj_LN294_M23 exhibit relatively higher antibiofilm activity than phage vB_KpnP_Henu1_3 against a hypervirulent *K. pneumoniae* strain (KL1) (Zhang et al., 2026). The high percentages of biofilm biomass reduction achieved by the three phages (65.56% to 99.96%) demonstrate their strong potential to destroy biofilms formed by classical and hypervirulent *K. pneumoniae* strains. This potential is achieved through a two-step mechanism. Biofilm is a bacterial community protected by a dense matrix of extracellular polymeric substances (EPS), proteins, and DNA. Initially, phages degrade this matrix and the protective capsule of the hypervirulent *K. pneumoniae* strains through the production of depolymerases (Fan et al., 2026; Sun et al., 2026). With the biofilm’s structure thus disrupted, phages gain direct access to the bacterial cells and can therefore initiate their lytic cycle. At this stage, the percentage of biofilm disruption depends on the phage’s biological characteristics (adsorption time, latency period, burst size, MOI, etc.). This explains the differences in percentages of biofilm disruption among the three phages studied. Phage Kp2Bj_LN294_M23 showed an increase in biofilm formation 48 h after treatment. The biofilm matrix of bacterial species generally consists of extracellular polymeric substances (polysaccharides, DNA, proteins, etc.) (Abdel-Razek et al., 2025; Wu et al., 2019). The increase in biofilm after 48 h of treatment could be explained by the bacteria’s response to phage pressure, leading to the activation of defense mechanisms and thereby increasing the production of extracellular matrix, which strengthens the biofilm structure. Alternatively, the partial destruction of the biofilm by the phages may release extracellular substances that act as nutrients, promoting the adhesion and growth of a new, denser biofilm. These reactions highlight the importance of phage cocktails in biofilm treatment. Thus, the phage cocktail consisting of Kp1Bj_HH11_M23, Kp2Bj_LN294_M23, and Kp10Bj_LN54_14 could be a very good candidate for preclinical and clinical testing in the treatment of infections caused by multidrug resistant, hypervirulent, and biofilm-producing *K. pneumoniae* strains.

For therapeutic purposes, it is important to maintain phage viability under various stress conditions (Islam et al., 2023). According to Bai et al. (2022), the industrial application of phages involves exposing them to common conditions with a temperature range of 4 to 40 °C and a pH ranging from 4 to 10 during production and processing. Kp1Bj_HH11_M23, Kp2Bj_LN294_M23, and Kp10Bj_LN54_14 remain stable within these temperature and pH ranges. All three phages retained partial activity under high-temperature conditions up to 70 °C, indicating that they are thermally stable and can be stored effectively. The pH tolerance of Kp1Bj_HH11_M23, Kp2Bj_LN294_M23, and Kp10Bj_LN54_14 ranges from 3 to 11, indicating that they can survive in the intestine and adapt to the microenvironments of different infection sites.

Although this study made it possible to isolate three *Klebsiella* phages with interesting characteristics (biological, genomic, and antibiofilm) locally in Benin, it remains limited in several respects. The tests conducted in our study do not allow us to state with certainty that phages Kp1Bj_HH11_M23, Kp2Bj_LN294_M23, and Kp10Bj_LN54_14 are the best candidates for preclinical or clinical trials in the treatment of human *K. pneumoniae* infections. Data are lacking on the synergistic effect of these phages in combination with an antibiotic, emergence of phage resistance, *in vivo* antibiofilm activity in an animal model, and *in vivo* safety assessment of these three phages. We will therefore continue our research to elucidate all these aspects not addressed in this study. Phage cocktails are recommended as a treatment to prevent the development of resistance to phages and to enhance lytic effects by expanding the phage-host range. We will also investigate the *in vivo* efficacy of phage cocktails with these three phages.

## 5 Conclusion

In summary, this study characterized the first *Klebsiella pneumoniae* phages isolated in Benin, Kp1Bj_HH11_M23, Kp2Bj_LN294_M23, and Kp10Bj_LN54_14, which belong to the family *Myoviridae* and the order Caudovirales. Kp1Bj_HH11_M23 and Kp2Bj_LN294_M23 are *Marfavirus*, while Kp10Bj_LN54_14 is a *Jiaodavirus*. These three novel phages exhibit lytic activity against more than 40 virulent and multidrug resistant clinical strains of *K. pneumoniae*. The genomes of these phages do not contain resistance or virulence genes, and showned that their are different of previous novel *Klebsiella* phage which infected multidrug resistant and hypervirulent *K. pneumoniae* strains. Their biological characteristics (rapid absorption, short latency periods, and the ability to release hundreds or even thousands of virions per infected cell during growth) and their biofilm disruption percentage further confirms their effectiveness. All of this, combined with their stability under challenging temperature and pH conditions indicates that they could be an potential candidates for production and therapeutic application. However, we must confirm this potential by continuing our research into aspects not addressed by this study–aspects that are important to clarify before moving on to preclinical or clinical trials for the treatment of infections caused by multidrug resistant and hypervirulent *K. pneumoniae* strains.

## Data Availability

The datasets presented in this study can be found in online repositories. The names of the repository/repositories and accession number(s) can be found in the article/Supplementary material.
